# Thromboembolic complications of intravenous immunoglobulin (IVIG) in an immunocompromised patient with Chronic Lymphocytic Leukemia: a case report

**DOI:** 10.1186/1757-1626-2-9078

**Published:** 2009-11-23

**Authors:** Cannon Milani, Samir M Dalia, Gerald A Colvin

**Affiliations:** 1 Warren Alpert School of Medicine at Brown University and Department of Medicine, Rhode Island Hospital, Providence, Rhode Island, USA; 2 Division of Hematology/Oncology, George 3, 593 Eddy Street Providence, Rhode Island 02903, USA

## Abstract

**Introduction:**

Infectious complications represent a major cause of morbidity and mortality in patients with chronic lymphocytic leukemia (CLL). The etiology is postulated to be secondary to aberrations in cell-mediated immunity, as well as to therapy-related immunosuppression. Hypogammaglobulinemia, which occurs in virtually all patients with CLL, may be profound and correlates with disease duration and stage. Intravenous immunoglobulin (IVIG) therapy has been used successfully to prevent and treat infections in this cohort of patients. However IVIG administration and treatment is not benign and should be used with caution given the potential manifestations of thromboembolic complications. High concentration and rapid infusion rate of the IVIG, as well as increased dose and osmolarity of the solution are thought to predispose to thrombotic events. Serum viscosity is the implicated mechanism for compromised blood flow and predisposition of high-risk patients to cardiovascular or cerebrovascular infarction. We report a case of IVIG related thromboembolic manifestations in a CLL patient, to highlight the importance of risk stratifying patients prior to treatment administration.

**Case presentation:**

We present a 55-year-old Caucasian man with CLL who presented to our clinic with neutropenic fevers following a cycle of chemotherapy. Laboratory parameters revealed hypogammaglobulinemia prompting IVIG administration. Shortly thereafter, he developed a massive cascade of thromboembolic phenomena precipitating his demise.

**Conclusion:**

The current consensus surrounding IVIG is that of a relatively safe treatment, with minor adverse effects such as hypertension, fever and chills, nausea, myalgias, or headache. However our report highlights the importance of proceeding with caution in the application of this therapy, as it's proclivity for thrombotic complications has not been fully elucidated in patients with underlying malignancies. Pre-existing thrombogenic risk factors should be carefully evaluated in patients undergoing treatment with IVIG. Clinical evaluation, with careful attention to vascular history and underlying co-morbidities can potentially unmask the high-risk patient where IVIG could be lethal.

## Introduction

The clinical application of immunoglobulins as a therapeutic agent dates back to more than a century when the first Nobel laureate, Emil Behring, observed that immune sera could ameliorate toxin-mediated diseases [1-3]. Immunoglobulins were first used for prophylaxis and treatment of infectious diseases. Initially preparations were injected intramuscularly with adverse limitation of painful myalgias and skin hypersensitivity reactions due to local proteolytic degradation. An intravenous application was not possible secondary to aggregates of purified immunoglobulins, leading to severe adverse reactions precipitated by activation of the complement cascade. The advent of new purification technologies allowed for the elimination of aggregates, and preparations of immunoglobulins for intravenous use became available [1-4].

In 1981 during treatment of two children with hypogammaglobulinemia and coincidental idiopathic thrombocytopenic purpura (ITP), physicians in Switzerland observed a reproducible increase in the platelet count following IVIG treatment. This clinical observation and follow up systemic investigations further intensified the widespread clinical use of IVIG as a potential immune modulatory agent [1].

Today, Intravenous immunoglobulin (IVIG) is used in a broad spectrum of autoimmune, inflammatory, and primary and secondary immunodeficiencies. The efficacy has been demonstrated in several control studies [5,6]. IVIG is a blood product prepared from the serum of between 1,000 and 15,000 donors per batch. The mechanism of action validated by in-vitro models is exerted by a combined effect on autoantibodies, complement activation, cytokines, and saturation of Fc receptors on tissue macrophages [1-3].

IVIG is considered a relatively safe treatment, which has contributed to its wide appeal. Most of the adverse events associated with IVIG are mild and transient. They commonly include fevers, chills, flushing, headaches, myalgias, blood pressure changes, tachycardia, and anaphylactic reactions, which are more pronounced in IgA-deficient patients [4-7]. Nevertheless, Brannagan et al. (1996) have reported a number of side effects. These were generally self-limited, but included serious complications such as deep venous thrombosis, pulmonary embolism, myocardial infarction, and stroke.

The mechanism of thromboembolic complications is postulated to be secondary to hyperviscosity, especially in patients having risk factors including advanced age, previous thromboembolic diseases, diabetes mellitus, hypertension, dyslipidemia, or those receiving high-dose IVIG at a rapid infusion rate [6,7].

Our patient in this case report manifested symptoms of expressive aphasia, weakness of this bilateral upper and lower extremities, and chest discomfort shortly following IVIG administration. As a result, we present this case report to demonstrate the importance of screening patients with underlying co-morbidities, especially malignant conditions and their higher predisposition for thromboembolism, prior to initiation of IVIG. Familiarity with adverse effects of IVIG can empower patients and physicians in their assessment of the risks and benefits prior to the use of this treatment.

## Case presentation

We present a case of a 55-year-old Caucasian man with a past medical history significant for Chronic Lymphocytic Leukemia (CLL) and Melanoma, diagnosed in 2006 and 2008 respectively, who presented to our Cancer Center complaining of a one-day history of persistent fevers and chills. The patient was visiting from out of state and was concerned about a potential infection. He had completed his 4th cycle of chlorambucil (40 mg per square meter, given orally every 28 days) for his CLL two weeks prior. The patient was status post surgical excision for a stage IIIA melanoma discovered in the left axilla with no further adjuvant treatment. He was scheduled to travel extensively over the course of the proceeding month and requested an additional course of antibiotics.

On review of systems, he noted progressive fatigue and anorexia. He had started a prescription for levofloxacin prior to the clinic visit. The patient had no known drug allergies. The patient denied any tobacco or alcohol use. He was employed in an office setting. There was no pertinent family history. The patient had a temperature of 38 degrees Celsius. Physical exam was remarkable for an enlarged spleen, approximately 3 finger-breadths below the costal margin. Laboratory parameters revealed a white blood cell count of 21,500 (normal 4500-11,000/mm3), a hemoglobin of 10.8 g/dl (normal 11-15 g/dl), and a platelet count of 170,000 (normal 150,000-400,000/mm3). His absolute neutrophil count (ANC) was 430 (normal 1,500 to 8,000/mm3). Further a quantitative immunoglobulin panel was consistent with hypogammaglobulinemia: IgG level was 309 L (normal 562-1585 mg/dl), IgM 9.0 L (normal 30-246 mg/dl), and IgA was 10 L (normal 72-372 mg/dl).

The patient was subsequently admitted to our hospital for evaluation and treatment of febrile neutropenia. We elected to administer 30 grams of IVIG (0.4 g/kg) as a means of enhancing his immunity in the context of recent exposure to chemotherapy and his hypogammaglobulinemia. The patient had previously tolerated multiple infusions of IVIG without any adverse reactions. We premedicated the patient with acetaminophen, hydrocortisone, and benadryl. Approximately 12 hours following the IVIG infusion, the patient began to complain of headaches, shortness of breath with accompanying chest pain, and weakness of his bilateral upper and lower extremities.

**Figure 1:**
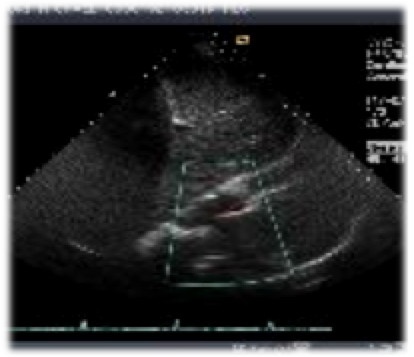
**Mural Thrombus in Left Ventricle**.

**Figure 2:**
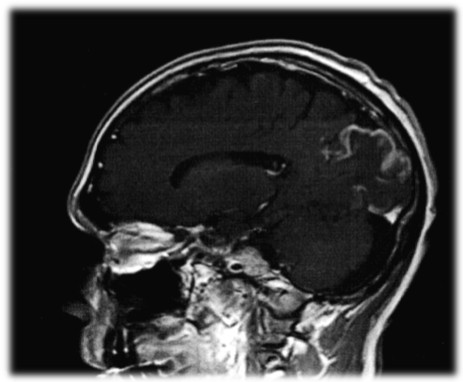
**Infarct Left Posterior MCA Distribution**.

**Figure 3:**
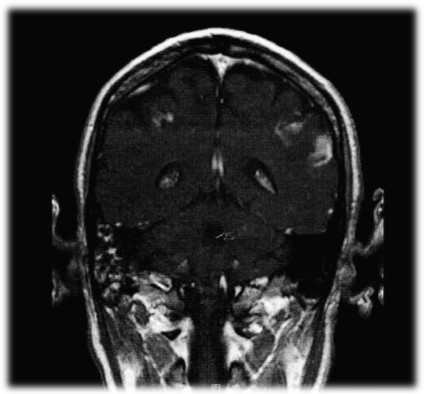
**Infarct Bilateral Parietal Lobes**.

**Figure 4:**
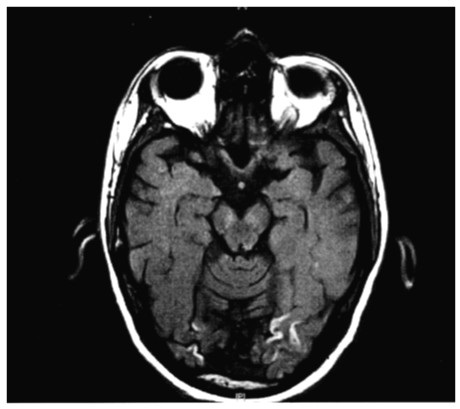
**Infarct Occipital Lobes**.

A complete cardiac and neurologic assessment was immediately initiated. The patient was found to have a myocardial infarction with a troponin elevation of 15.2 ng/ml and 13.3 ng/ml (normal 0.00-0.15 ng/ml) for the first two sets of his cardiac enzymes respectively, with a clearance of the enzymes by his third set. The EKG revealed ST-T wave changes consistent with myocardial ischemia in the inferior and anterolateral regions. An echocardiogram was consistent with a mural thrombus (Figure 1) visualized in the left ventricular apex. There was mild global left ventricular hypokinesis, with inferior and inferolateral wall motion abnormalities consistent with myocardial infarction. In addition, an MRI of the brain (Figures 2, 3 and 4) delineated evolving cerebral infarcts seen within the left posterior MCA distribution (Figure 2), bilateral high parietal loops (Figure 3), and bilateral occipital lobes (Figure 4). There was no evidence of significant mass effect or hemorrhagic transformation.

Furthermore, the patient began to experience a change in his mental status. A Neurology consult was obtained and the assessment attributed his waxing and waning consciousness to the continued watershed infarcts that were present. Nevertheless an EEG study was performed which delineated periodic lateralized epileptiform discharges consistent with seizure like activity. The patient was subsequently placed on intravenous levetiracetam with no improvement in his mental status. Shortly thereafter he had increasing respiratory requirements, and the decision was made to forgo intubation at the request of the family secondary to the patient's pre-determined request to never be placed on a ventilator.

## Conclusion

To date, IVIG has more often been considered a safe medication, with minor adverse effects such as hypertension, fever and chills, nausea, myalgia or headache. However, with the wider use of IVIG in the treatment of hypogammaglobulinemia associated malignancies, and various autoimmune disorders, the potential for severe sideeffects such acute renal tubular necrosis, aseptic meningitis, anaphylaxis in patients with IgA deficiencies, and thrombotic manifestations need to be considered.

In concordance with previous reports [1-10], we documented a close temporal association between a cardiovascular and cerebrovascular insult secondary to IVIG infusion. Our patient suffered a myocardial infarction (MI) and stroke within 24 hours of the IVIG infusion. Furthermore, whereas rapid infusion protocols have previously been identified as a possible thomboembolic risk factor, our patient received standard doses of IVIG at conventional infusion rates.

The pattern of stroke in multiple vascular territories (Figures 2, 3 and 4) on MRI imaging portends to a thromboembolic mechanism of stroke, rather than occlusion of atherosclerotic cerebral arteries or small vessel disease. A possible mechanism of stroke is the introduction of clotting factors and/or vasoactive cytokines that would trigger the thrombotic cascade and lead to in situ thrombosis and embolism. Alternatively, elevated plasma serum viscosity has been proposed as a likely mechanism by some [3]. Our case report indicates that patients should be monitored closely for these types of adverse events during any period of IVIG therapy. In patients presenting with predictive factors of thrombotic complications, IVIG should be prescribed cautiously, after judiciously weighing the risk-benefit considerations.

## Consent

In terms of obtaining consent for publication, all reasonable attempts to gain consent have been made. The patient's next of kin reside overseas and as a result establishing contact has been unsuccessful. We declare that the patient is anonymous and we have no reason to believe that the patient or their family would object to this publication. We briefly mentioned the potential of writing up a case report when the patient was still alive, and the family felt that if it would benefit medical practice, they would support our efforts.

## Competing interests

The authors declare that they have no competing interests.

## Authors' contributions

CM and SD analyzed and interpreted the patient data in regards to the administration of IVIG therapy and its subsequent complications. CM, SD, and GC were major contributors in writing the manuscript. All authors read and approved the final manuscript.
